# Supplementary material for: Collaborative intelligence in AI: Evaluating the performance of a council of AIs on the USMLE
Source: PLOS Digit Health. 2025 Oct 9;4(10):e0000787. doi: 10.1371/journal.pdig.0000787 (PMC12510544; doi:10.1371/journal.pdig.0000787)
Supplement: S2 File — (ZIP) [file pdig.0000787.s002.zip › S2 Data Raw data files containing unprocessed question inputs and LLM outputs including transcripts of deliberations/STEP3_Question-127-1_Type-MC-NJ.docx]

**Facilitator** **[Round 1]:** A 20-year-old woman comes to the office because of a 2-day history of nasal congestion, runny nose, and nonproductive cough that has made it difficult for her to sleep. She has not had fever. Her brother with whom she resides also has similar symptoms. The patient's medical history is significant for mild persistent asthma for which she takes inhaled fluticasone- salmeterol twice daily and albuterol as needed. She has been using her albuterol more frequently during the past 2 days without much effect. During this time, her peak expiratory flow rates have been 70% to 80% of her baseline. Vital signs today are temperature 37.2°C (99.0°F), pulse 70/min, respirations 20/min, and blood pressure 110/70 mm Hg. Physical examination discloses inflamed nasal mucosa with clear discharge. The posterior oral pharynx is erythematous. Auscultation of the lungs discloses rare expiratory wheezes. Which of the following is the most appropriate pharmacotherapy for this patient's cough? (A) Codeine (B) Diphenhydramine (C) Guaifenesin (D) Ipratropium bromide (E) Prednisone

**AI_E:** 1) RESTATING THE QUESTION: Essentially, the question here is trying to ask which drug would be the most effective in handling this 20-year-old woman's cough, which is a part of her symptoms including a runny nose and nasal congestion that has lasted 2 days. This woman takes fluticasone-salmeterol for her asthma and has been needing albuterol increasingly over the last couple days. Technical Terms: - Fluticasone-Salmeterol: A combination drug which contains a corticosteroid (Fluticasone) that reduces inflammation and a bronchodilator (Salmeterol) that relaxes muscles in the airways. - Albuterol: A bronchodilator that helps with breathing issues, such as is often needed with asthmatic individuals. 2) PLANNING: The assumption could be made that this woman's cough and other symptoms are due to a cold, or perhaps even allergies. However, her brother is also experiencing similar symptoms, suggesting that this may not be the case. The symptoms also occur concurrently with a redirection of her peak expiratory flow rates (70%-80% from baseline), indicating a worsening of her asthma. 3) CLINICAL REASONING: Thus, her symptoms could be affecting her asthma. This is evidenced by the fact that she has been using her albuterol prescription more frequently lately, to little effect. This suggests that her asthmatic symptoms have worsened, likely due to her present cold-like symptoms. As a result, a change in medication seems to be in order. 4) CONCLUDING: In this case, the most effective pharmacotherapy likely wouldn't be focused on her cough or cold-like symptoms per se, but rather her asthma which seems to be the overarching issue at hand. Her asthma doesn't respond well to her current treatment anymore, which indicates that an adjustment of her asthma treatment is needed. 5) RESPONSE: Therefore, to answer the question, the most appropriate pharmacotherapy for her cough isn't a cough suppressant or something similar. Instead, a better choice might be Prednisone (E), a corticosteroid. This would reduce inflammation in her airways, consequently alleviating her nonproductive cough and addressing her worsening asthma.

**AI_B:** RESTATING THE QUESTION: The question is about a 20-year-old woman who is presenting with upper respiratory tract symptoms like a runny nose, nasal congestion, and a nonproductive cough. She has been experiencing this for two days and also having difficulty sleeping due to these symptoms. Her brother has similar symptoms, and she has a history of mild persistent asthma, which isn't controlled adequately over the past two days as evident by the increased usage of rescue inhaler, albuterol, and decreased peak expiratory flow rates. The physical examination suggests inflammation in the nasal mucosa and mild wheezing in the lungs. The question is asking which drug would be the most suitable to relieve her cough. PLANNING: The immediate assumption could be that her symptoms, including cough, are due to respiratory infection, probably a common cold (as her brother also has similar symptoms) which might have exacerbated her underlying asthma. However, we could challenge this by saying that her symptoms could be a flare-up of her asthmatic condition triggered by an allergen, stress, exercise, or viral infection. Thus, treating her underlying asthma may help manage her cough. CLINICAL REASONING: As an asthmatic patient (already on controller medications), she has inflammation in her bronchial tubes that makes them hyper-responsive. An upper respiratory viral infection (common cold) may have triggered this asthmatic exacerbation, causing bronchospasms and thus her cough. In this step, control of her asthma should be the main focus, for which systemic corticosteroids might be needed given inadequate response to albuterol and decreased peak flow rates. CONCLUDING: Thus, controlling her sinus symptoms and cough isn't the only goal. Instead, the proper management of her asthma symptoms should be the main focus, considering her decreased peak flow rates. Relief of her asthma symptoms will subsequently alleviate her cough. RESPONSE: The most appropriate pharmacotherapy for this patient's cough is (E) Prednisone, which is a systemic corticosteroid. Systemic corticosteroids are often used in step-up therapy for those patients with asthma not adequately controlled by or worsening on initial therapy. They help reduce the inflammation in the bronchial tubes, easing the bronchospasm and allowing easier breathing, thus relieving symptoms, including cough.

**AI_D:** RESTATING THE QUESTION: The question asks what would be the most effective medication to treat a nonproductive cough in a 20-year-old woman with a short history of upper respiratory symptoms and a longer history of mild persistent asthma. Here, "nonproductive cough" refers to a cough that does not expel any mucus or phlegm, and "peak expiratory flow rates" are a measure of how fast a person can exhale, which can indicate if their airways are narrowed as in conditions like asthma. PLANNING: There are few assumptions we can make. The lack of fever and the presence of similar symptoms in her brother suggest a viral infection. Despite her increase in albuterol usage, she still exhibits respiratory symptoms, indicating that her asthma is not entirely under control. Since her cough is nonproductive, it may also exacerbate her current asthma state, as both coughing and asthma affect the airways. Assessing these possibilities, it would be clinically important to both manage her cough and control her asthma given her past medical history. CLINICAL REASONING: Since she presents with respiratory symptoms without fever, has a family member with similar symptoms, and her oral pharynx is inflamed, a viral upper respiratory infection is a possible diagnosis. These infections often exacerbate asthma symptoms, leading to increased use of rescue medications, such as albuterol. That she's been using albuterol without relief suggests the inflammation from her upper respiratory infection isn't being entirely managed by her current medication regimen, necessitating additional treatment. Rather than treating her cough symptomatically (as with codeine, guaifenesin, or diphenhydramine), her asthma exacerbation needs to be directly addressed. CONCLUDING: The medication that best addresses both her viral upper respiratory infection-induced cough and her uncontrolled asthma is a drug that has an anti-inflammatory effect in airways. Neither a centrally acting antitussive (e.g., Codeine) nor an expectorant (e.g., Guaifenesin) would adequately treat the patient's underlying issue. Diphenhydramine, an antihistamine, may dry respiratory secretions, worsening the problem. Ipratropium bromide, a bronchodilator, only provides symptomatic relief. Hence, the most appropriate medication would be Prednisone. RESPONSE: The most appropriate pharmacotherapy for this patient's cough is Prednisone (E). Prednisone, a systemic corticosteroid, will help decrease the inflammation in her airways caused by the viral infection, improving her cough and helping control her asthma.

**AI_C:** 1) RESTATING THE QUESTION: We have a 20-year-old female patient who is exhibiting symptoms of an upper respiratory tract infection such as a runny nose, nasal congestion, and a non-productive cough. She additionally has a history of mild persistent asthma and her symptoms are not being adequately controlled by her current medications, causing her peak expiratory flow rates to drop. The main aim here is identifying the medication that would best alleviate her cough. 2) PLANNING: We first need to examine her symptoms and diagnose her condition correctly. Her symptoms suggest she may be experiencing an upper respiratory infection, possibly a common cold. It's essential to consider whether the cough is due to the infection, an asthma exacerbation, or both. Her increased albuterol use and lower peak expiratory flow rates indicate that her asthma isn't in optimal control, which could be exacerbated by the current viral illness. A re-evaluation of her pharmacotherapy might be needed. 3) CLINICAL REASONING: In the context of her URTI and asthma, we're primarily concerned with managing her cough. Codeine (an opioid antitussive), Diphenhydramine (an antihistamine), Guaifenesin (an expectorant), Ipratropium bromide (an anticholinergic bronchodilator), and Prednisone (a corticosteroid) all have potential roles to play. Codeine, while effective for cough suppression, can have negative side-effects and isn't recommended for routine use in acute cough. Diphenhydramine can help if the cough is allergy-induced, but it doesn't seem to be the case here. Guaifenesin is typically used for productive coughs, while hers is non-productive. Ipratropium bromide, being a bronchodilator, could address the component of cough due to bronchoconstriction in an asthma flare. Prednisone, a systemic corticosteroid, might be an effective choice if the patient's asthma symptoms are exacerbated and not responding to her current medication. 4) CONCLUDING: Considering both her current symptoms of an URTI and the evidence of asthmatic exacerbation, the best approach would be to primarily address her underlying poor asthma control while managing her URTI symptoms. 5) RESPONSE: For the current situation, "Prednisone" (E) would be the most appropriate choice. It could help reduce the inflammation during her current asthma exacerbation, which should help in controlling the cough. We could then consider the other pharmacotherapies for better management of her overall health in future consultation.

**AI_A:** RESTATING THE QUESTION: The question pertains to the best treatment option for a young woman experiencing nasal congestion, runny nose, and a bothersome nonproductive cough for two days. The patient, who has a history of mild persistent asthma, has been increasingly using her as-needed albuterol, but to little effect. Her peak expiratory flow rates have dropped to 70% - 80% of her usual levels. Her physical examination revealed inflammation in her nasal mucosa with a clear discharge, and the back of her oral pharynx appears reddened. There are few expiratory wheezes noted on lung auscultation. The appropriate pharmacological treatment is to be decided amongst options such as Codeine, Diphenhydramine, Guaifenesin, Ipratropium bromide, and Prednisone. PLANNING: The primary assumption here is that the young woman's symptoms are related to respiratory irritation, more specifically an upper respiratory tract infection (URTI). The fact that her brother also shares her symptoms indicates a possible transmittable etiology. However, given her history of asthma and the reduced effectiveness of her albuterol, an alternate assumption could be that she is also experiencing an exacerbation of her asthma, possibly triggered by her current upper respiratory infection. Thus, the pharmacotherapy chosen should primarily address her most distressing symptom (the nonproductive cough) and also take into consideration potential asthma exacerbation. CLINICAL REASONING: The patient is currently on fluticasone-salmeterol as a controller medication for asthma and albuterol as a rescue inhaler. However, her peak expiratory flow rates have decreased despite increased use of albuterol, a short-acting beta-2 agonist (SABA). This signifies that her asthma may not be well-controlled amidst her current URTI episode. The medications listed have different mechanisms of action and indications. Codeine and Diphenhydramine are centrally-acting cough suppressants, Guaifenesin is an expectorant, Ipratropium bromide is an anticholinergic bronchodilator, while Prednisone is a glucocorticoid. As her cough is nonproductive, an expectorant like Guaifenesin may not be beneficial. Ipratropium bromide, though a bronchodilator, is usually not first-line for asthma exacerbations. Prednisone, a systemic corticosteroid, would be a reasonable choice given the indication of a mild asthma exacerbation. Codeine, although strong, could risk dependency and is more suited for severe chronic cough while her cough is substrate less. Diphenhydramine is more effective for allergic conditions, and its sedative properties might make it useful for relieving her sleep problems due to cough. CONCLUDING: Prioritizing her declining respiratory function due to her underlying asthma along with her nonproductive cough, the selection of appropriate medication needs to be targeted at possibly curtailing an impending asthma exacerbation while providing symptomatic relief. RESPONSE: Based on her present symptoms and past medical history, the most suitable pharmacotherapy for her nonproductive cough and asthmatic condition would be the systemic corticosteroid, Prednisone (option E). Prednisone can assist in managing an asthma flare-up, decreasing inflammation in the airways, reducing respiratory distress, and as an adjunct, could potentially ease her cough. Nonetheless, the patient should be further evaluated, and her asthma control should be reassessed regularly.

**Facilitator** The consensus among the AI responses is that the most appropriate pharmacotherapy for the 20-year-old woman's nonproductive cough and worsening asthma symptoms is Prednisone (E). The woman's symptoms suggest an upper respiratory tract infection, possibly a common cold, which may have exacerbated her underlying asthma. Her increased use of albuterol and decreased peak expiratory flow rates indicate that her asthma is not well-controlled. The medications listed have different mechanisms of action and indications. Codeine and Diphenhydramine are centrally-acting cough suppressants, Guaifenesin is an expectorant, Ipratropium bromide is an anticholinergic bronchodilator, while Prednisone is a glucocorticoid. Given her nonproductive cough and evidence of an asthma exacerbation, Prednisone, a systemic corticosteroid, is the most suitable choice. It can help manage an asthma flare-up, decrease inflammation in the airways, reduce respiratory distress, and potentially ease her cough. However, the patient should be further evaluated, and her asthma control should be reassessed regularly.
